# Association between age and outpatient clinic arrival time: myth or reality?

**DOI:** 10.1186/s12913-018-3057-2

**Published:** 2018-04-02

**Authors:** Kashif Waqar Faiz, Espen Saxhaug Kristoffersen

**Affiliations:** 1 Department of Neurology, Akershus University Hospital, PO Box 1000, 1478 Lørenskog, Norway; 2 Health Services Research Center, Akershus University Hospital, Lørenskog, Norway; 3 Department of General Practice, Institute of Health and Society, University of Oslo, Oslo, Norway

**Keywords:** Outpatient clinic, Age, Patient flow, Non-attendance, Unpunctuality

## Abstract

**Background:**

Non-attendance and late arrivals diminish patient flow in outpatient clinics. On the other hand, patient earliness may also be undesirable. Physicians often experience that older patients are more punctual than younger patients, and often they come excessively early. The aim of this study was to determine whether an association between age and outpatient clinic arrival time could be established or not, i.e. to find out if it is a myth or a reality.

**Methods:**

Prospective descriptive study performed at a neurological outpatient clinic. Data were collected from all scheduled appointments during an eight-week period. Variables included were age, gender, appointment time, arrival time, no-shows, appointment type, need for assistance and if it was an early or late appointment. Outcomes were unpunctuality (early and late arrivals) and non-attendance.

**Results:**

Of 1353 appointments, non-attendance rate was 9.5 and 5.1% were late arrivals. Median age increased with increased patient earliness (*p* <  0.001). Younger age (*p* = 0.007) and new referrals (*p* = 0.025) were associated with non-attendance.

**Conclusions:**

The intuition of an association between age and outpatient clinic arrival time was confirmed, thus it is a reality that older patients attend their appointments more frequently and have better punctuality than younger adults. This age effect in outpatient clinics should be considered when developing future simulation models and intervention studies.

## Background

Clinic scheduling is important for the planning of an optimal patient flow in outpatient clinics. Because of the limited resources available in the health care system and the demands for increased efficiency and productivity, non-attendance and late arrivals cause economic cost and clinical inefficiency. Non-attendance prolongs waiting lists and causes physician idle time. Late arrivals disrupt the outpatient clinic schedules, result in delays for other patients and can cause shorter appointments or overtime as physicians fall behind schedule. Turning away tardy patients is a possibility, but this can increase the risk of worsened health for the patient. On the other hand, patients arrive early more often than late, and patient earliness may also be undesirable, since it creates overcrowding in the waiting area and potentially reduces the patient logistics, which in turn can be a challenge to both the patient and the front-desk staff [1]. Previous research on outpatient clinic scheduling and patient flow has mainly focused on late arrivals [2–4] and no-shows/ non-attendance [5–10]. Age and gender have previously been suggested as potential factors for non-attendance [7–11]. However, very few studies have investigated factors associated with patient earliness. With increased focus on well-organized outpatient clinics to reduce both the number of and the length of in-hospital admissions, investigating new factors such as patient earliness at outpatient clinics are important, as these factors may be associated with reduced effectiveness and logistics.

The authors of this study have experienced that older patients are more punctual than younger patients and often they come excessively early. Furthermore, this intuition is shared by other colleagues. Thus, the aim of this study was to determine whether an association between age and outpatient clinic arrival time could be established or not, i.e. to find out if it is a myth or a reality.

## Methods

### Study design and population

This prospective study was performed at the outpatient clinic, Department of Neurology, Akershus University hospital, Norway. The hospital’s catchment area consisted of a mixed population (urban and rural) of approximately 490,000 inhabitants (about 10% of Norway’s population). Furthermore, the department of Neurology (both inpatient and outpatient clinic) is the largest specialised neurological department in Norway.

The study population consisted of all scheduled appointments for adults ≥ 18 years of age during an eight-week period (October and November 2013). All data were collected by the front-desk staff on arrival. The study had no exclusion criteria.

Patients attending the neurological outpatient clinic are either new referrals mainly referred by their general practitioner (GP) or follow-ups. A senior consultant decides on urgency upon evaluating the clinical information, and medical secretaries subsequently send an appointment schedule to the patient. Furthermore, many patients with chronic neurological diseases have regular specialist follow-up appointments, e.g. botulinum toxin therapy every third month for patients with dystonia and spasticity, and natalizumab treatment for patients with multiple sclerosis every fourth week, as well as other neurological diseases such as Parkinson’s disease, epilepsy, migraine and myasthenia gravis.

### Outcomes

The variables collected were age, gender, appointment time, arrival time, no-shows, appointment type (new referral or follow-up appointment), need for assistance (accompanied by a caregiver or not on show-up) and appointment time (if it was an early [8.00–11.59 AM] or late [12.00–4.00 PM] appointment).

Non-attendance was defined as failure to attend the appointment without notification.

Unpunctuality was defined as appointment time minus arrival time (in minutes) [12]. Negative values reflected the waiting time for early arrivals, and positive values reflected the delay time for late arrivals.

### Statistics

Categorical variables are presented as frequencies and percentages. Continuous variables are presented as medians and interquartile ranges (IQR) or means and standard deviations (SD).

Multivariable linear regression analysis was performed to analyse variables associated with early arrival time. Gender, age, appointment type, appointment time and need for assistance were included in the multivariable model.

Multivariable logistic regression analyses were performed on non-attendance and patient lateness as binary outcomes variables. Gender, age, appointment type and appointment time were included in the model. Odds ratios (OR) are presented with 95% confidence intervals (CI). The Kruskal-Wallis test was used to analyse the association between age and unpunctuality, where earliness was categorized into four different groups; < 0 min, 0–15 min, 16–30 min and > 30 min.

Potential non-linear effects of age on the three outcome measures (early arrivals, late arrivals and no-shows) were investigated using regression models with splines.

The statistical analyses were performed using SPSS version 20.0 (SPSS Inc., Chicago, IL) and 5% was the level of significance. R version 3.4.0 was used to fit the regression models with splines.

### Approvals, ethics and data security

The study was considered a quality assurance project, which, according to Norwegian law on medical research, does not require an approval by the Regional Ethics Committee, nor a written patient consent. The local Data Protection Authorities approved the study. Data were anonymized and secured on a research server at Akershus University Hospital. The authors had full access to the study data.

## Results

A total of 1353 appointments were scheduled in October and November 2013 and thus included in the study. The median age was 50.0 years (IQR 40.0–65.0) (mean 51.4 years, SD 16.4), and 60.2% were women. Almost one out of four needed assistance from a caregiver. More data on population characteristics are shown in Table 1.

**Table 1:** Study population characteristics (*N* = 1353)

Gender	
Females (%)	815 (60.2)
Males (%)	538 (39.8)
Age, median (IQR)	50.0 (40.0–65.0)
Age, mean (SD)	51.4 (16.4)
Referral type	
New referrals (%)	449 (33.2)
Follow-up appointments (%)	904 (66.8)
Appointment time	
Early (08.00–11.59 AM) (%)	810 (59.9)
Late (12.00–4.00 PM) (%)	543 (40.1)
Attendance	
Show-ups (%)	1225 (90.5)
No-shows (%)	128 (9.5)
Need for assistance (*)	
No (%)	898 (73.3)
Yes (%)	327 (24.2)
Punctuality (*)	
Early arrivals (%)	1162 (94.9)
Late arrivals (%)	63 (5.1)
Early arrivals, waiting time, minutes, median (IQR) (**)	15.0 (7.0–24.3)
Early arrivals, waiting time, grouped, minutes (**)	
0–15 min (%)	657 (56.5)
16–30 min (%)	335 (28.8)
> 30 min (%)	170 (14.6)
Late arrivals, delay time, minutes, median (IQR) (***)	5.0 (2.0–10.0)

*IQR* interquartile range, *SD* standard deviation

(*) From show-ups only (*N* = 1225)

(**) From early arrivals (*N* = 1162)

(***) From late arrivals (*N* = 63)

In all, 33.2% of the appointments were new referrals and 66.8% follow-ups. Non-attendance rate was 9.5% (128 appointments). Among new referrals, the non-attendance rate was 12.0% (54/449) and among follow-ups 8.2% (74/904). Of the 1225 attending patients, 63 (5.1%) were late arrivals. For early arrivals, the median waiting time was 15.0 min (IQR 7.0–24.3). One out of six of the early arrivals showed up more than 30 min before their appointment.

**Fig. 1:**
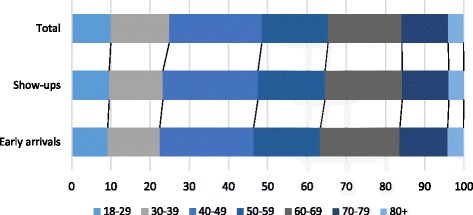
Age distribution for the total study population, show-ups and early arrivalss

**Fig. 2:**
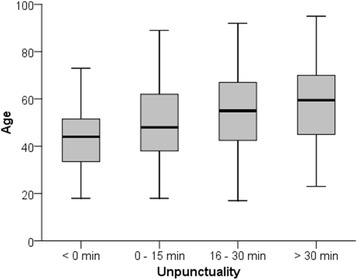
Age distribution in four unpunctuality groups; < 0 min = late arrivals; > 0 min = early arrivals

**Fig. 3:**
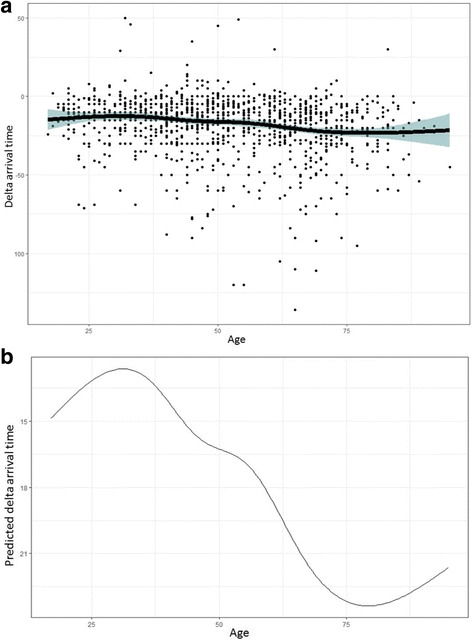
**a**, **b** Non-linear effects of age on arrival time using regression models with splines

Figure 1 shows the age distribution for the total study population, show-ups and early arrivals. Figure 2 shows a boxplot of the age distribution in four unpunctuality groups. The boxplot shows increasing median age with increased waiting time (*p* <  0.001). Using multivariable linear regression analysis (Table 2), there was a significant association between older age and increased patient earliness (*p* <  0.001). Figures 3a and b show the non-linear effects of age on arrival time using regression models with splines (*p* <  0.001).

**Table 2:** Multivariable linear regression analysis, variables associated with early arrival time (*N* = 1162; patients with non-attendance and late arrival excluded from the analysis)

	Multivariable, *p* value (*)	Beta coefficient (95% confidence interval) (*)	Multivariable, *p* value (**)	Beta coefficient (95% confidence interval) (**)
Gender	0.507	− 0.69 (− 2.72–1.34)	0.480	−0.73 (− 2.76–1.30)
Age	< 0.001	0.18 (0.12–0.24)	< 0.001	1.70 (1.08–2.32)
Appointment type	0.180	1.44 (− 0.67–3.55)	0.171	1.47 (− 0.64–3.59)
Appointment time	0.180	1.38 (− 0.64–3.41)	0.186	1.370 (− 0.66–3.40)
Need for assistance	0.613	−0.58 (− 2.81–1.66)	0.627	− 0.56 (− 2.80−1.30)

(*) Age as a continuous variable

(**) Age categorized into 10-year groups according to Fig. 1

In the early arrival group 316/1162 (27.2%) patients needed assistance and were accompanied by a caregiver on show-up. In a subgroup multivariable linear regression analysis excluding patients who needed assistance (*N* = 846), older age (*p* <  0.001) and new referrals (*p* = 0.024) were associated with early arrival time, while gender and appointment time were non-significant (*p* = 0.347 and *p* = 0.423, respectively).

**Fig. 4:**
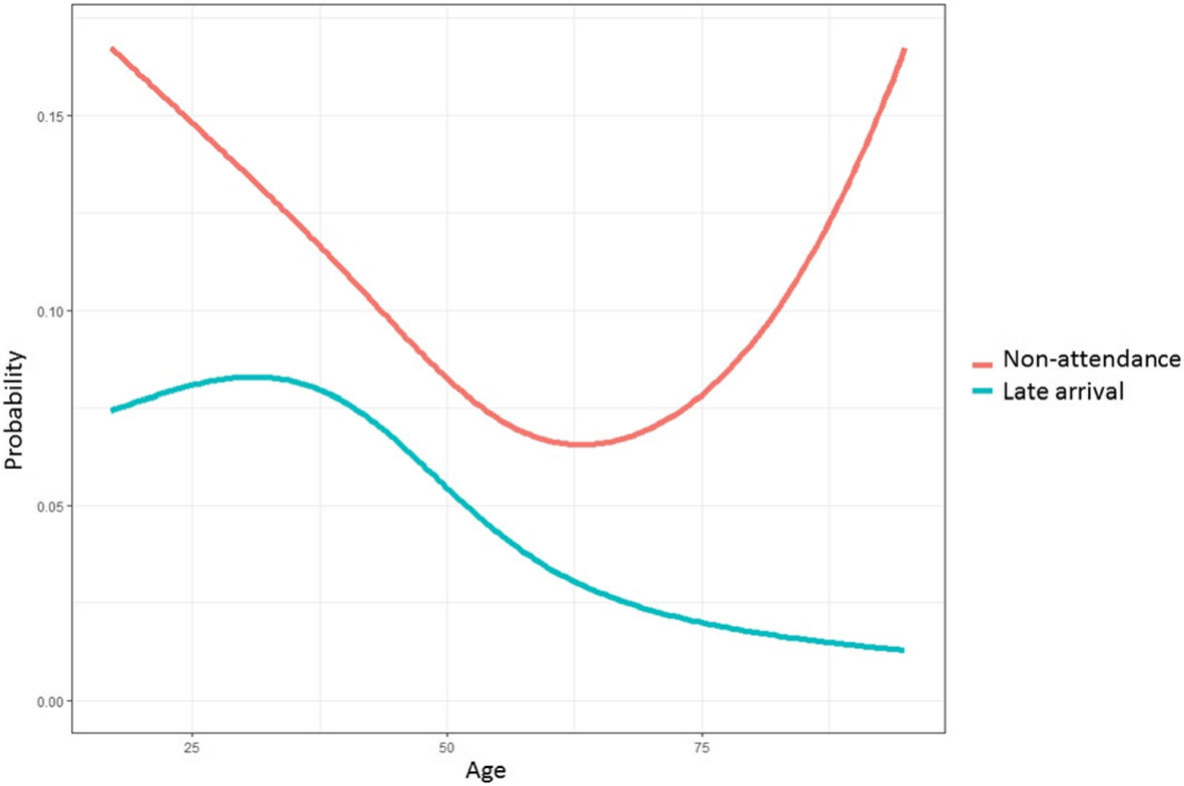
Non-linear effects of age on non-attendance and late arrival using regression models with splines

Using multivariable logistic regression analysis (Table 3), younger age (*p* = 0.007; OR 0.98, 95% CI 0.97–0.99) and new referrals (*p* = 0.025; OR 0.65, 95% CI 0.45–0.95) were associated with non-attendance. The median age of show-ups was 51.0 (IQR 40.0–65.0) years, and of no-shows 45.0 (IQR 34.3–62.8) years. Late arrival (Table 4) was also associated with younger age (*p* <  0.001; OR 0.97, 95% CI 0.95–0.99) in a multivariable logistic regression model. The median age of early arrivals was 51.0 (IQR 40.0–65.3) years, and of late arrivals 44.0 (IQR 33.0–52.0) years. Figure 4 shows the non-linear effects of age on late arrival (*p* <  0.001) and non-attendance (*p* = 0.011) using regression models with splines.

**Table 3:** Multivariable logistic regression analysis, variables associated with non-attendance (*N* = 1353)

	Multivariable, *p* value (*)	OR (95% confidence interval) (*)	Multivariable, *p* value (**)	OR (95% confidence interval) (**)
Gender (male)	0.910	0.98 (0.67–1.43)	0.902	0.98 (0.67–1.42)
Age	0.007	0.98 (0.97–0.99)	0.006	0.85 (0.76–0.95)
Appointment type (new referral)	0.025	0.65 (0.45–0.95)	0.023	0.65 (0.45–0.94)
Appointment time (early appointment)	0.260	1.24 (0.85–1.79)	0.256	1.24 (0.86–1.80)

(*) Age as a continuous variable

(**) Age categorized into 10-year groups according to Fig. 1

**Table 4:** Multivariable logistic regression analysis, variables associated with late arrival (*N* = 1225; patients with non-attendance excluded from the analysis)

	Multivariable, *p* value (*)	OR (95% confidence interval) (*)	Multivariable, *p* value (**)	OR (95% confidence interval) (**)
Gender (male)	0.403	0.80 (0.48–1.35)	0.415	0.86 (0.48–1.36)
Age	< 0.001	0.97 (0.95–0.99)	< 0.001	0.74 (0.63–0.88)
Appointment type (new referral)	0.474	1.23 (0.70–2.17)	0.501	1.22 (0.69–2.14)
Appointment time (early appointment)	0.155	1.45 (0.87–2.43)	0.153	1.45 (0.87–2.43)

(*) Age as a continuous variable

(**) Age categorized into 10-year groups according to Fig. 1

## Discussion

This simple study shows a significant association between older age, early arrival time and lower rates of non-attendance in a neurological outpatient clinic, thus verifying the intuition that older patients are more punctual than younger patients.

A novel finding in the present study was that there is a significant association between older age and increased patient earliness, also when adjusted for gender, appointment type, appointment time and need for assistance in order to get to the outpatient clinic. Age-related earliness could be related to that older individuals have more free time, they are more conscious about their health, or a shift in the younger part of the community regarding attitude or respect toward punctuality and attendance in general. From the hospital perspective, early arrival is much better than late arrival. However, as many as 15% of the patients showed up more than 30 min before their appointments. This degree of earliness increases the risk of overcrowded waiting areas, less overview for the hospital staff and might lead to lower patient satisfaction. Because of limited space, more patients may risk standing while waiting for their appointment, which may be problematic for elderly and chronically ill patients. We speculate that attendance approximately 15 min before the appointment is a reasonable earliness where pros and cons are levelled out for patients and the health care system. In this regard, the median waiting time in the present study was accurate. An intervention tailored towards avoiding too early arrival seems less expedient and may very well lead to the contrary, which is even less favourable. Furthermore, at least a partial explanation for very early arrivals could be the transport arrangement and traffic situation.

In this study, the non-attendance rate was 9.5%, which is consistent with previous studies from different medical settings showing non-attendance rates ranging from 2 to 30% [5, 7, 8, 13–16]. Comparison between these studies should be done with caution due to very varying settings and study populations. However, in the UK, which has a similar health care system as in Norway, the non-attendance has been reported to be about 12% [17]. Our results could be extrapolated to about 800 cancelled appointments due to non-attendance only at our outpatient clinic each year, which lead to direct loss of revenue of approximately 80,000 Euros, in addition to several indirect costs. Non-attendance is a major health care system challenge in terms of social costs of unused resources such as staff, ward capacity and misuse of other patients’ time. In addition, the hospital and health region authority receive a lower reimbursement than budgeted. Because of the large numbers, even a small reduction in no-shows would save a significant cost.

It is possible to speculate that patients who have not attended their appointments in the past may be more likely to repeat the behaviour [18], and thus further worsening the total waiting list situation. Long waiting time before appointment is not only a health risk, but is related to reduced patient satisfaction [19].

In one study from general practice, the most common reasons for missing appointments were forgetfulness, appointment at an inconvenient time, family commitments and illness [6]. There are somewhat conflicting results whether non-attendance is more common in new referrals than for follow-ups [11, 20, 21]. In the present study, non-attendance was more common for new referrals than for follow-up appointments. This is important to address as the duration of new appointments in our outpatient clinic is longer (60 min) compared to regular follow-ups (30 min), making the non-attendance for new appointment even more costly and wasteful for both the hospital and the society. Inadequate communication between the referring physician and the patient has been suggested as a reason for more frequent non-attendance in new referrals [22, 23]. Possible explanations may be that the patient does not know why he or she is referred; the patient may not be aware of the seriousness of a disorder, or may even be referred to different hospitals or departments without the knowledge that these different appointments concern different organ systems or disorders. The latter may be an even bigger problem in the future with an increasing proportion of elderly patients with multi-morbidity combined with a more fractioned and sub-specialised health care system. Furthermore, a long time period between referral and appointment time has been suggested as a risk factor for non-attendance [21, 24].

In the present study, older age was associated with lower non-attendance, which is comparable to findings from other studies [7, 8, 11, 13]. In a Swiss Internal Medicine outpatient clinic [7], the non-attendance rate was 15.8%, and these patients were significantly younger than the controls (38.4 versus 43.6 years). Similarly, in a study from a multidisciplinary outpatient clinic including about 13,000 appointments [8], the no-show rate decreased for every increase in age quartile, and for every 1-year increase in age, the absolute no-show rate decreased by 2,4%. Possible explanations for this age effect suggested in the article were family and employment engagements among younger patients, and increased health problems among elderly patients, thus increasing the tendency to keep appointments [8]. Also in our study, it is reasonable to assume that elderly patients have more severe and advanced neurological conditions and therefore have a higher need to see a physician, thus the higher attendance-rate.

Although non-attendance has been the major focus of most studies concerning schedule planning and improvement of patient flow, some intervention [3, 8, 25] and modelling and simulation [2, 26] studies exist with promising results. Most randomized controlled trials on appointment reminders by telephone and text messaging (short message service [SMS]) have shown that non-attendance rates are significantly lower in the intervention groups compared to control groups with an average reduction of non-attendance of about 9% [27]. One study showed a non-significant reduction [28]. Furthermore, both telephone reminders and SMS one day before the scheduled appointment were cost-effective [25, 27, 29]. In contrast, only four out of sevens studies testing postal reminders were positive with an average reduction of non-attendance of 7.6% [27]. Letting the patients book their own appointments has also been suggested as a possible option to reduce non-attendance. A primary care study investigated the effect of giving the patients a copy of the referral letter, however, this did not affect the attendance rates [17].

In an intervention study from a pain clinic in Baltimore, USA [3], 9.6% of the patients arrived after the appointment time, but this lateness rate was reduced to 4.6% after an intervention where patients were informed that tardy patients would not be seen and would be rescheduled. Furthermore, mean unpunctuality changed from - 20.5 to - 25.0 min, i.e. patient earliness increased. Additionally, simulations revealed that reducing patient unpunctuality reduces delays. Unfortunately, the impact of age on patient earliness was not evaluated [3]. In a study on late arrival from a paediatric outpatient clinic [4], 10% of about 65,000 visits were late arrivals. The odds of late arrival were increased for patients who spoke English (compared to Spanish), Medicaid or no insurance and late arrival on their previous appointment. Age was not included in the analysis. In our study, the late arrival rate of 5.1% was somewhat lower compared to other studies, and late arrival was associated with younger age. In contrast to our findings, Hang et al. [4] showed that patients were most likely to be late to early morning appointments.

As non-attendance and unpunctuality remain important issues with large implications for patients, physicians and society, it is of uttermost importance to reduce such behaviour. New interventions may be tailored towards patients, GPs and hospital systems and should be based on the existing evidence. The age effect should be considered when developing future simulation models and intervention studies. Flexible scheduling with a mix of older and younger patients may be a possibility, alternatively selectively overbooking a mix of younger patients. Models focusing on a mix of different age groups in order to avoid the daily variation on non-attendance and late arrivals would also be beneficial, and may increase physician and staff satisfaction.

Some limitations need to be addressed. This was a single-centre study from a single speciality. Characteristics and behaviour of patients with neurological conditions may differ from patients attending outpatient clinics from other specialities. The results can thus not necessarily be generalized. However, the age and gender distribution was most probably similar to that found in most Norwegian general medicine outpatient clinics. It is not possible to exclude that some patients may have been included more than once, however, it is extremely rare with more than one appointment over a two-month period (except for the few patients with multiple sclerosis treated with natalizumab every fourth week). The patients’ travel distance from home to the hospital was not collected, neither if the patients had to arrange the transport to hospital. The actual time the appointment started, i.e. physician delay, was not recorded. In addition, information was not collected on patients’ reasons for non-attendance or late arrival. No interventions were initiated.

## Conclusions

In conclusion, we demonstrate that older patients attend their appointments more frequently and have better punctuality than younger adults. Patients who require assistance will obviously arrive early because of coordination and logistics issues, and new patients who are unfamiliar with the facility will also tend to be early. The elderly who tend to be retired will also be early, while younger, mobile patients will tend to not be early because of competing priorities, and returning patients who already know the check-in processes will understandably tend to not arrive early.

This age effect in outpatient clinics should be considered when developing future simulation models and intervention studies. Flexible scheduling with a mix of older and younger patients may be a possibility, alternatively selectively overbooking younger patients [11]. Reduced patient unpunctuality will improve patient flow and thus, outpatient clinic performances with a better outcome for both the individual patient and the society.

## Abbreviations

CI: Confidence interval
GP: General practitioner
IQR: Interquartile range
OR: Odds ratio
SD: Standard deviation
SMS: Short message service
